# Transcriptome sequencing of black grouse (*Tetrao tetrix*) for immune gene discovery and microsatellite development

**DOI:** 10.1098/rsob.120054

**Published:** 2012-04

**Authors:** Biao Wang, Robert Ekblom, Todd A. Castoe, Eleanor P. Jones, Radoslav Kozma, Erik Bongcam-Rudloff, David D. Pollock, Jacob Höglund

**Affiliations:** 1 Population Biology and Conservation Biology, Department of Ecology and Genetics, Evolutionary Biology Centre, Uppsala University, Norbyvägen 18 D, 75236 Uppsala, Sweden; 2 Evolutionary Biology, Department of Ecology and Genetics, Evolutionary Biology Centre, Uppsala University, Norbyvägen 18 D, 75236 Uppsala, Sweden; 3 Department of Biochemistry and Molecular Genetics, University of Colorado School of Medicine, 12801 17th Avenue, Aurora, CO 80045, USA; 4 Department of Animal Breeding and Genetics, Swedish University of Agricultural Sciences, PO Box 7084, 75007 Uppsala, Sweden

**Keywords:** bird, spleen, RNA-seq, immune genes, major histocompatibility complex, microsatellites

## Abstract

The black grouse (*Tetrao tetrix*) is a galliform bird species that is important for both ecological studies and conservation genetics. Here, we report the sequencing of the spleen transcriptome of black grouse using 454 GS FLX Titanium sequencing. We performed a large-scale gene discovery analysis with a focus on genes that might be related to fitness in this species and also identified a large set of microsatellites. In total, we obtained 182 179 quality-filtered sequencing reads that we assembled into 9035 contigs. Using these contigs and 15 794 length-filtered (greater than 200 bp) singletons, we identified 7762 transcripts that appear to be homologues of chicken genes. A specific BLAST search with an emphasis on immune genes found 308 homologous chicken genes that have immune function, including ten major histocompatibility complex-related genes located on chicken chromosome 16. We also identified 1300 expressed sequence tag microsatellites and were able to design suitable flanking primers for 526 of these. A preliminary test of the polymorphism of the microsatellites found 10 polymorphic microsatellites of the 102 tested. Genomic resources generated in this study should greatly benefit future ecological, evolutionary and conservation genetic studies on this species.

## 2. Introduction

The ever-decreasing cost of next-generation sequencing has extended new research avenues previously restricted to model organisms to a wide range of other species [1–3]. One such research avenue is transcriptome sequencing, which allows efficient large-scale gene discovery and marker development [4–7]. This approach is an effective way to generate genomic resources for non-model species, including those important for ecological and evolutionary biology research [8–10]. Once a relatively large number of gene sequences have been generated, they can be used to identify fitness-related genetic loci that may interact with the organismal phenotype, and thus provide an insight into how evolutionary processes have shaped the genotype and phenotype of these organisms [11,12]. The transcriptome is composed of many functional sequences that can be annotated using known gene information from related model species (where these are available), making it a particularly efficient way to identify genes that may be related to the fitness of an organism in the environment [13]. Transcriptome sequencing can additionally be used for digital gene expression profiling analysis [14,15], construction of microarrays and development of large sets of genetic markers, such as single nucleotide polymorphisms or microsatellites [7,16–18].

Because of its early availability, together with its longer read lengths, Roche 454 sequencing is a commonly used next-generation sequencing platform in transcriptome sequencing projects of non-model organisms. Its longer sequence reads facilitate high-quality de novo assembly of the transcriptome in the absence of a characterized reference genome [9]. With ever-increasing numbers of genome sequencing projects, ecologists can now often make use of a related complete genome to annotate the assembled transcripts from their species of interest and then target particular loci that might be of interest, including those related to the organism's fitness in the environment [10].

The black grouse (*Tetrao tetrix*) is a galliform bird species that has been well studied from an ecological perspective, including studies focused on behavioural ecology, sexual selection and the evolution of the lek mating system [19–21]. It is also a focal species for conservation genetics [22], and its long-term persistence is threatened by increasing fragmentation of natural populations across its range [23]. The genetic effects of inbreeding and genetic drift on isolated grouse populations have been estimated using a number of markers and major histocompatibility complex (MHC) class II sequences [24–28]. Thus, the availability of a characterized transcriptome for the black grouse, and identification of a large set of genes and genetic markers, would be transformative in facilitating research progress on a number of important topics.

In this study, we conducted transcriptome sequencing of spleen tissue of a male black grouse using the Roche 454 GS FLX sequencing platform. The main aim of the project was to characterize the transcriptome of this wild species and to identify genes that might be relevant to the genetic basis of fitness variation. We used gene annotations from the chicken (*Gallus gallus*) to annotate the grouse transcriptome because grouse and chickens are closely related galliform birds [29]. We chose the spleen, an important immune organ in mature vertebrate animals, as the source RNA to enrich for the discovery of immune-related genes, which are generally considered to encode a large amount of fitness-related loci with high variations among individuals [30]. We also identified microsatellite (simple sequence repeat) markers from the assembled grouse transcripts and tested for polymorphism of these expressed sequence tag (EST)-based microsatellites in several individuals. In addition to developing more microsatellite markers for this species, we also evaluated the efficiency of this microsatellite development strategy.

## 3. Material and methods

### 3.1. Sampling, library preparation and 454 sequencing

The black grouse individual used for the 454 sequencing was a male collected by a licensed hunter near Uppsala, Sweden. The fresh spleen tissue of the sample was immediately isolated and stored in RNAlater (Ambion). Total RNA was extracted using TRIzol (Invitrogen) following the manufacturer's protocol. The quality and quantity of the RNA was estimated using a nono-RNA chip run on the Agilent Bioanalyzer 2100 (Agilent).

Approximately 5 μg of total RNA was enriched for mature mRNA transcripts using three successive rounds of purification with Oligo dT^25^ beads (PureBiotech), precipitated using linearized acrylamide, sodium acetate and ethanol, and analysed using a Bioanalyzer pico-RNA chip. The mRNA was reverse transcribed with random heptamers and modified oligo-dT primers (5′-/Phos/NNNNNNN-3′ and 5′-/Phos/TTTTTVN-3′) in a 2 : 1 ratio, using the SuperScript III reverse transcriptase kit (Invitrogen). The remaining RNA was digested using RNAse A and RNAse H and purified using RNA Clean beads (Ambion). Two pairs of double-stranded (with single-stranded overhang) adapter oligonucleotides were directionally ligated onto the existing synthesized first strand using T4 DNA Ligase (Invitrogen). Adapter oligonucleotide sequences were: adapter-A (5-prime adapter), oligo A-prime 5′-NNNNNNCTGATGGCGCGAGGGAGG-dideoxyC-3′ and oligo A 5′-GCCTCCCTCGCGCCATGAG-3′; and adapter-B (3-prime adapter) oligo B 5′-biotin-GCCTTGCCAGCCCGCTCAGNNNNNN-phosphate-3′, and oligo B-prime 5′-phosphate-CTGAGCGGGCTGCAAGG-dideoxyC-3′. Ligation products were purified using RNA Clean beads three successive times and then with streptavidin beads (PureBiotech). Samples were then melted from the streptavidin beads using 0.1 M NaOH and precipitated (as mentioned earlier). Completed libraries were quantified and checked for appropriate size distribution using a nano-DNA chip on a Bioanalyzer.

The resulting cDNA library was sequenced in two partial runs of the 454 GS FLX sequencing instrument with Titanium XL reagents and 70 × 75 mm PicoTiterPlates (PTPs). In one run, the PTP was physically divided into 16 regions and two of these were occupied by the grouse cDNA sample. In the second run, the PTP was physically partitioned into eight regions, and one of these was occupied by the grouse cDNA sample.

### 3.2. Assembly, annotation, Gene Ontology analysis and identification of genes with immune function

The sequencing reads were assembled into contigs using Newbler (GSAssembler v. 2.0.01, 454 Life Sciences) with default parameters (minimum overlap length = 40, minimum overlap identity = 90%, contig length threshold = 100 bp). All of the adapters were entered into the GSAssembler trimming database before the assembly was performed. For the sequencing reads that were not assembled (singletons), we used the SeqClean program (http://compbio.dfci.harvard.edu/tgi/software/) to screen and remove the low-quality reads, to trim the adapters and poly A/T stretches, and to filter-out reads shorter than or equal to 200 bp. To evaluate the sequencing quality, we used BLAT [31] to map all the contigs and filter-passed singletons to the repeats-masked chicken genome (WUGSC 2.1/galGal3), which was downloaded from the UCSC genome browser.

Ensembl chicken protein sequences were extracted via BioMart (http://www.biomart.org/biomart/martview/) with the parameters of Ensembl gene 61, *Gallus gallus* genes (WASHUC2). The homology search was carried out by BLASTX (NCBI BLAST 2.2.24+) at an *e*-value criterion of 1 × 10^–10^. Only the best BLAST hit records were kept in the downstream analysis. For the sequences that did not have BLAST hits, we extracted all the bird proteins from NCBI Taxonomy ‘Aves (birds)’ entry and performed an additional BLAST search for them using BLASTX and an *e*-value threshold of 1 × 10^–10^. The remaining sequences from the second BLAST attempt were used to perform a homology search against the NCBI non-redundant database using BLASTX and *e*-value threshold of 1 × 10^–10^.

All the transcripts, including contigs and singletons, that had positive BLAST hits in the earlier-mentioned annotation analysis were selected to perform the Gene Ontology (GO) analysis. The selected sequences were imported into Blast2GO program and were used in a BLAST search against the NCBI non-redundant database with an *e*-value criterion of 1 × 10^–10^ [32]. Mapping and annotation of the GO terms for the BLAST results were performed using the default parameters of Blast2GO. A final GO graph was generated, which summarized the distribution of the GO level 2 terms. The same GO analysis was performed against Ensembl chicken transcripts and overrepresentation of biological processes (BPs) terms was analysed using GOstat with default settings [33].

To emphasize the identification of the genes with immune function, the GO level 2 term ‘immune system process’ and all its children terms were extracted from the GO chicken ‘BP’ database. These immune GO terms were then converted to Ensembl identifiers and were used to extract protein sequences from the Ensembl chicken database via BioMart. The homology search was performed using BLASTX at an *e*-value criterion of 1 × 10^–10^ and only the best hit records were retained. The results were summarized using all levels of the extracted immune GO terms.

### 3.3. Identification and validation of microsatellites

All the contigs and filter-passed singletons were used to screen for microsatellites. The screening was performed in Msatcommander v. 1.0.8 [34], which could identify the microsatellites and simultaneously design the PCR primers using the inbuilt program Primer v. 3 [35]. We screened only perfect di-, tri-, tetra- and penta-nucleotide repeats and used a threshold of six repeats for di-nucleotides and four repeats for the others. As we planned to test a large set of the designed primers, we followed an economical strategy by tagging the primers with universal tags M13 (5′-GGAAACAGCTATGACCAT) or CAG (5′-CAGTCGGGCGTCATCA) [36,37], which subsequently linked to a universal fluorescent-labelled tag. Designing of the primers and selection of the tag were performed automatically by Msatcommander.

We experimentally tested 102 microsatellites using three criteria to select which to test. We first selected those that had annotation information; that is, the sequences used to design the primers could be annotated using the chicken genome. In this way, we knew which genes the microsatellites were associated with. Second, we balanced the primers among the different chromosomes. Third, we gave priority to di-nucleotide and tri-nucleotide primers, which are likely to have higher mutation rates [38].

The DNA samples that were used to test the microsatellite primers were from two individuals from Jyväskylä, Finland, one individual from Jämtland, Sweden and one individual from Kristiansand, Norway. The microsatellite primers were synthesized with one standard and one tagged primer. Two fluorescently labelled universal primers (M13-FAM and CAG-HEX) that bound to the tag were also used. For the subsequent PCR reaction, the primers were used in the ratio of 1 tagged primer : 10 simple primer : 10 universal dye-labelled primer [37]. PCRs were run using the Qiagen Type-it Microsatellite kit under the recommended conditions. Amplified products were genotyped on a Megabace 1000 automatic sequencer (Amersham Biosciences, Buckinghamshire, UK), and allele sizes were scored using the MegaBACE fragment profiler v. 1.2 (Amersham Biosciences 2003).

## 4. Results and discussion

### 4.1. 454 sequencing and assembly

**Figure 1.**
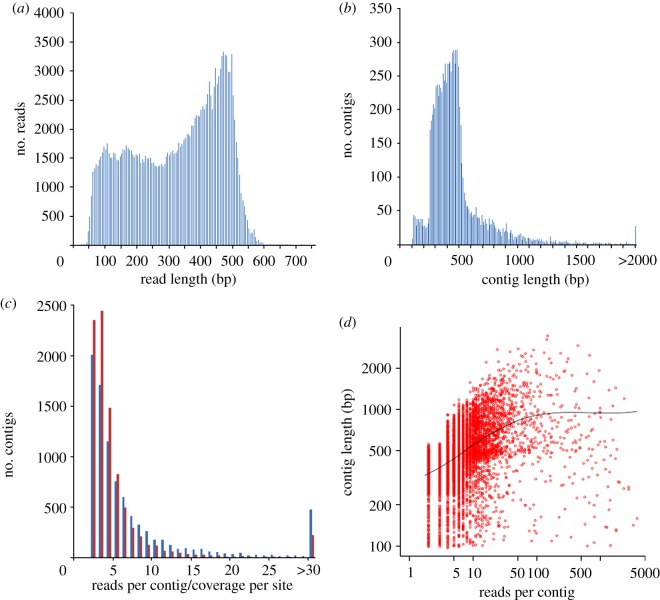
A summary of sequencing and contig assembly results. (*a*) Length distribution of the pre-process 454 quality-filter-pass reads. (*b*) Length distribution of assembled contigs. Contigs larger than 2000 bp are binned at the end of the *x*-axis. (*c*) Distribution of reads per contig (blue) and coverage per nucleotide site (red). Contigs with more than 30 reads are binned at the end of the *x*-axis. (*d*) Density scatterplot showing relationship between reads per contig and contig length. The black line represents the trend of the contig length with increasing reads per contig. Both the *x*- and *y*-axes are presented on a log scale.

In total, we sequenced one 1/8 and two 1/16 454 GS FLX Titanium runs, nearly the equivalent of 1/4 of a run. The raw sequences were deposited in the NCBI short read archive under accession number SRA036234. After adapter trimming and quality filtering, we retained a total of 182 179 reads, with a mean length of 320 ± 140 bp (table 1; figure 1*a*). Of these, 153 065 (84.0%) reads were assembled into 9035 contigs with a length threshold of 100 bp. The mean length of the contigs was 470 ± 250 bp (figure 1*b*), with 2276 of the contigs being larger than 500 bp. The mean number of reads per contig was 18.81, and the average contig coverage per nucleotide site was 10.01 (figure 1*c*). For the trimmed and cleaned reads that were not assembled (the singletons), only those longer than 200 bp were included in downstream analysis. There are 15 794 such singletons and their mean length is 370 ± 90 bp. To generally confirm the quality of the singletons and the contigs, we mapped all of them to the chicken genome (WUGSC 2.1). In sum, 19 497 of 24 829 sequences (78.5%), including the contigs and the size-filtered singletons, could be mapped to the chicken genome. The failure of the rest sequences could be due to the fact that the chicken genome itself has not been well completed—for example, many of the microchromosomes are under-represented, and many complicated regions with copy number variations are absent [39]. Also, by the nature of the identification process, truly novel transcribed sequences are generally ignored [40].

**Table 1.** Summary of sequencing and assembly results.

number of reads	182 179
read length (bp)	320 ± 140
number of reads assembled	153 065
percentage of reads assembled	84
number of contigs	9035
contig length (bp)	470 ± 250
reads per contig	18.81
coverage per nucleotide site	10.01
number of singletons	15 794
singleton length (bp)	370 ± 90

There was a clear positive correlation between contig length and reads per contig for those with fewer than 50 reads per contig (Pearson's correlation *r* = 0.56, d.f. = 8698, *p* < 0.0001), but when the coverage exceeded this threshold the correlation with contig length essentially vanished (Pearson's correlation *r* = 0.05, d.f. = 333, *p* < 0.0001; figure 1*d*). This correlation is expected because there is a greater probability of having greater numbers of reads for longer contig. On the basis of this empirical relationship, contigs with more than 50 reads are likely to represent full-length assemblies of transcripts, while many of the contigs with fewer than 50 reads may represent incompletely assembled transcripts.

### 4.2. Annotation and Gene Ontology analysis

Homology searches were performed using the Ensembl chicken database (WASHUC2). On the basis of this, 12 593 (50.7%) transcripts, including contigs and singletons, had BLAST hits with an *e*-value no larger than 1 × 10^–10^, and this resulted in hits involving 6852 homologous chicken genes (table 2). There are two possible reasons that the identified genes were fewer than the total number of transcripts with BLAST hits. One reason is that some transcripts may not have been completely assembled (thus having multiple non-overlapping contigs or singletons). A second reason might be that that some sequences were from different transcript isoforms of the same gene. The total number of chicken homologues identified (6852) represent 38.2 per cent of the entire Ensembl chicken gene set, having hits on most chromosomes and the mitochondrial genome, and the number of the genes discovered has a strong correlation with the chromosome length (Pearson's correlation *r* = 0.97, d.f. = 6850, *p* < 0.0001).

**Table 2.** Summary of annotation results.

homology search using Ensembl chicken database (WASHUC2)	
number of transcripts (contigs + singletons)	24 829
number of transcripts used	12 593
number of genes discovered	6852
additional homology search using NCBI bird proteins	
number of remaining transcripts	12 236
number of transcripts used	1150
number of genes discovered	910

For the 12 236 transcripts without BLAST hits to the Ensembl chicken database, we performed additional homology searches by using the bird protein set from the NCBI non-redundant database. From this secondary search, 1150 transcripts had BLAST hits to a total of 910 bird genes. Of these, 608 of the bird genes were from other bird species, including chicken, turkey (*Meleagris gallopavo*), quail (*Coturnix coturnix*) and zebra finch (*Taeniopygia guttata*), which matched 284 of the 910 genes. For the remaining transcripts with no BLAST hits up to this point, we performed homology searches against the entire NCBI non-redundant database. This resulted in 192 transcripts with BLAST hits. Among these hits, we found 51 genes that matched malaria parasites (*Plasmodium* spp*.*), plausibly indicating that the black grouse is a wild host for bird malaria.

**Figure 2.**
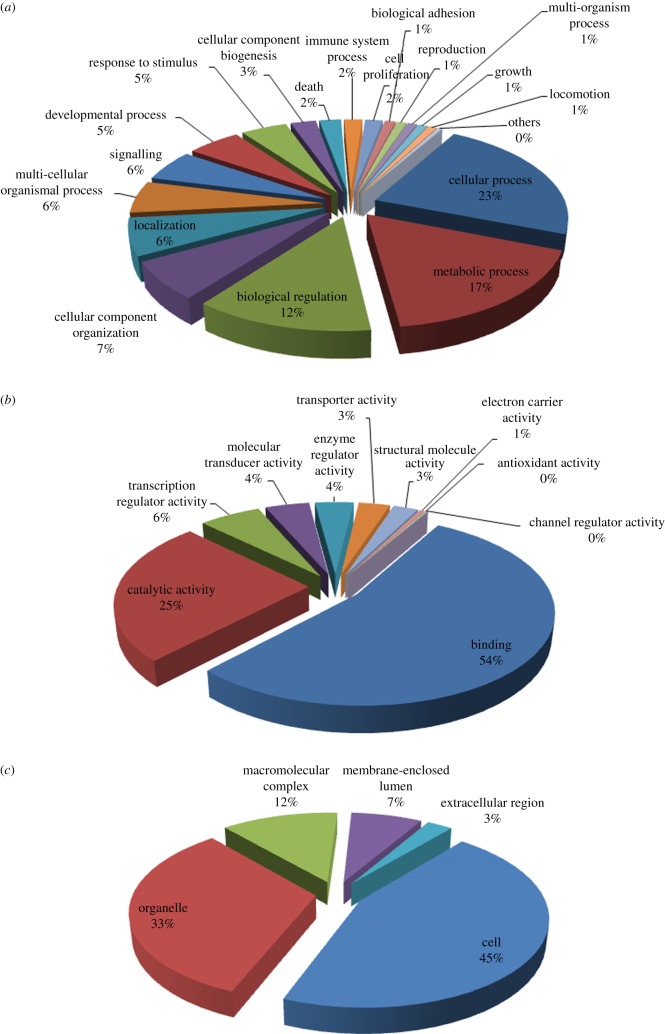
Distribution of the Gene Ontology (GO) functional categories. The transcripts of the black grouse spleen were classified into GO categories of (*a*) biological process (BP), (*b*) molecular function (MF) and (*c*) cellular component (CC) on the basis of GO second level terms.

To better understand the functions of the newly identified black grouse genes, we took transcripts that had positive BLAST hits in the earlier-mentioned homology searches and imported them into the Blast2GO suite [32], and then used GO (www.geneontology.org) terms to classify them into functional categories. The results included 21 286 GO terms for BP, 16 602 GO terms for molecular function (MF) and 13 993 GO terms for cellular components (CCs), representing a broad range of different biological activities and functions. To present results in a more accessible fashion, we used GO level 2 terms to summarize GO categories (figure 2). Within the BP category, the top three assigned terms were cellular processes, metabolic processes and biological regulation, representing 52 per cent of all terms. Immune system processes constituted 2 per cent of the BP terms and were overrepresented compared with GO annotation of Ensembl chicken genes (Fisher's exact test, *p* = 0.0312). Within the MF category, 54 per cent of terms were protein binding and 25 per cent of terms were catalytic activity. Of the CC category, 59 per cent of the terms were related to the cell and 24 per cent were related to organelles.

### 4.3. Identification of genes with immune function

**Figure 3.**
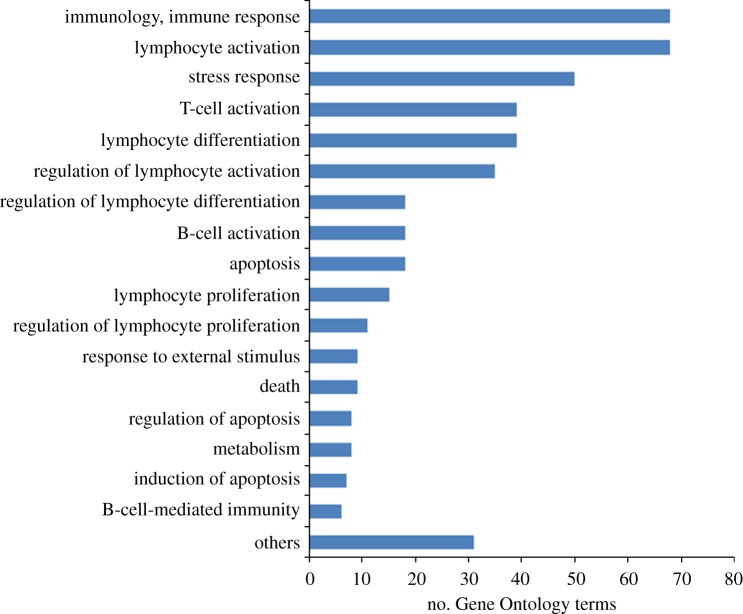
Distribution of the immune-related GO terms. The transcripts with immune functions were assigned to all levels of immune-related GO terms. The top 17 of the represented terms are shown and the rest are binned at the bottom.

To further focus on the identification of genes with immune function, we extracted 257 GO terms, including the GO level 2 term ‘immune system process’ and all its children terms, for chicken. On the basis of these GO annotations, we extracted 523 chicken immune-related gene sequences from Ensembl. Another stand-alone BLAST search was performed with our transcripts against these immune-related chicken genes, where we found 564 black grouse transcripts that matched to 308 homologous immune genes in the chicken. These black grouse transcripts had a mean length of 449 ± 236 bp. Our black grouse transcript set, although relatively modest in size, appears to contain homologues to a majority (58.9%) of all the extracted chicken immune genes. This percentage is notably higher than the percentage of all Ensembl chicken genes with matches in the grouse spleen in this study (38.2%). We expect that this is probably due to the highly enriched immune function of the spleen tissue we sequenced. The annotated immune genes are involved in a diversity of immune functions, including lymphocyte activation, T-cell activation, B-cell activation and various immune regulations (figure 3).

The annotated set of black grouse immune-related genes included homologues to several of the MHC genes located on chicken chromosome 16 (table 3), the micro-chromosome where the ‘minimal-essential MHC’ of the chicken is located [41]. Both the MHC class I and class IIB genes, which play an important role in the immune response by presenting the digested antigen fragments on the surface of the cell to T cells, were identified. The average depth of sequence coverage (per site) of these reconstructed transcripts was 16.05 and 23.11, respectively; these levels of coverage are notably high compared with the mean overall coverage of 10.01 (Student's *t*-test, *p* < 0.0001). The entire peptide-binding region (PBR; also called antigen recognition site or antigen presenting site) of MHC class I and part of the PBR region of class IIB genes were among the identified genetic regions. Those regions are thought to be among the most variable loci in vertebrate genomes (particularly across individuals), and are therefore useful for future ecological and evolutionary studies [42,43].

**Table 3.** Chromosome 16 MHC genes identified.

transcript ID	transcript length (bp)	Ensembl protein ID	gene symbol	gene description
contig01538	1384	ENSGALP00000000233	*BF*	MHC class I antigen B-F major heavy chain
contig08853	249	ENSGALP00000000193	*BLB*	MHC class II beta chain
contig08938	353			
contig08968	402			
contig02821	491	ENSGALP00000000213	*BMA1*	MHC class II M alpha chain
contig02432	490	ENSGALP00000040419	*BMA2*	MHC class II M beta chain 2
contig02454	2014	ENSGALP00000000211	*BRD2*^a^	bromodomain containing 2
FZYUT3M04XU4W0	441	ENSGALP00000000182	*BLec1*	B-lec C-type lectin-like receptor
contig01331	603	ENSGALP00000000202	*TAPBP*	tapasin precursor
contig08153	323			
contig02591	807	ENSGALP00000040428	*TAP2*	transporter associated with antigen processing 2 fragment
FZYUT3M04XTXK7	252			
contig01293	1697	ENSGALP00000000170	*GNB2L1*	guanine nucleotide-binding protein subunit beta-2-like 1
FZYUT3M04YN4L0	434	ENSGALP00000019549	*TRIM7*^a^	tripartite motif protein 7

^a^Genes that are curated based on a double-check of the NCBI RefSeq database.

### 4.4. Microsatellite development

We identified 1300 microsatellites from the 24 829 transcripts in total, including contigs and filter-passed singletons. Tri-nucleotide microsatellites were the dominant repeat type found (61.9%), followed by di-nucleotides (25.9%), tetra-nucleotides (8.5%) and penta-nucleotides (3.6%) (table 4). It is likely that tri-nucleotides were so prevalent because they can remain in coding regions without causing reading frame shifts. We set 450 bp as cut-off for the length of the PCR product, although a higher cut-off could be used. Among all the microsatellites, it was possible to create PCR primers for 526 (40.5%) loci. For these 526 microsatellites, we mapped the transcripts within which they were found onto Ensembl chicken genes (WASHUC2). A total of 306 (58.2%) could be mapped (table 4; electronic supplementary material, table S1). From these microsatellites, we selected 30 di-nucleotides, 70 tri-nucleotides and 2 tetra-nucleotides to test whether they amplified correctly and were polymorphic. The tested microsatellites included loci that mapped to chicken chromosomes 1–27 and Z (see electronic supplementary material, table S2).

**Table 4.** A summary of microsatellites identified.

	number of loci identified	number of loci with primers designed	number of loci with annotated information	number of loci tested
di-nucleotides	337	91	34	30
tri-nucleotides	805	388	258	70
tetra-nucleotides	111	33	8	2
penta-nucleotides	47	14	6	0
total	1300	526	306	102

Of the 102 screened microsatellite loci, 23 were readily amplified via PCR, 10 of which were polymorphic across the samples we tested (table 5). The polymorphic microsatellites were from chromosome 1 (BG03, BG04, BG07, BG14, BG15, BG21), chromosome 2 (BG26), chromosome 3 (BG29), chromosome 14 (BG78) and chromosome 19 (BG94). Overall, the rate of the amplified microsatellites was lower compared with some published projects using genome (rather than transcriptome) sequencing [6,44]. This may be because the genomic DNA can contain introns that do not appear in the transcribed DNA (the transcriptome). These introns (invisible in the transcriptome) may disrupt the amplification of some of the designed microsatellite primers; it would take only a small intron between the designed primer pairs for the microsatellite to fail to amplify in the screening. It is also possible that the published literature under-represents studies with relatively low microsatellite discovery rates. Six of 30 (20%) di-nucleotide microsatellites and four of the 70 (6%) of tri-nucleotide microsatellites were polymorphic. The lower diversity of the tri-nucleotide microsatellites may be because they were from exons, which only have very low mutation rates, while the di-nucleotides might be from untranslated regions and therefore more variable [45].

**Table 5.** A summary of polymorphic microsatellites.

locus	repeat motif	primer sequence (5′–3′)	number of alleles	allele size (bp)	related gene
BG03	(AGC)	F: GCACTCTTCACTAGCAGCCC	3	146–164	DNA repair protein RAD52 homolog
		R: CAAGCAGGGTCAGAGCATTG			
BG04	(AC)	F: GGGTCTCTTGCTTCCTTGAC	2	219–221	ATP-binding cassette, sub-family C, member 9 isoform SUR2A
		R: TTAAACTTCATGCTCACACGC			
BG07	(AT)	F: CAGTTACAGCAAGGACAGAGC	2	127–141	putative uncharacterized protein, UniProtKB/TrEMBL Acc. Q5ZM27
		R: GGGAGCCAACAAGAATAAACTG			
BG14	(AT)	F: ACAGCGCCTTCCCTATATCC	2	146–149	claudin domain containing 1
		R: TGACCAAACTTTGCCGGAAG			
BG15	(AG)	F: ACAGACACAGAAAGCATCCC	3	312–316	amyloid beta A4 protein
		R: TGCTGTAACACAAGTAGATGCC			
BG21	(ACG)	F: AACATCACGCCGTTTCACTG	2	124–127	probable ATP-dependent RNA helicase DDX10
		R: AAGCCGCGTTCCAAACAC			
BG26	(AC)	F: TGACAGCCTGGGAAGTATGC	2	264–268	C-type lectin domain family 3, member B
		R: CACCAGTGGCTCTTTGATGC			
BG29	(AGG)	F: CCAGCTTTCATGACCACGTC	3	136–142	alpha-L RNA-binding motif superfamily
		R: TCAGTACTCTCTCTGCGGAAC			
BG78	(AGG)	F: TCTTCAGGGCTTTCTCAGGG	2	234–240	ABC transporter-like
		R: CATGAAACCTGTCAGCGTGG			
BG94	(AC)	F: TGAACCTGAGAAGGCAAAGG	3	130–148	sarcoplasmic/endoplasmic reticulum calcium ATPase 3
		R: AGCATCAGGGTGAGGTGTC			

For the polymorphic microsatellites, we obtained a fairly low number of alleles, although this is propably because they were typed for only four individuals, all from Scandinavia. It is therefore possible that some of the seemingly monomorphic microsatellites would be polymorphic if more geographically distant populations were tested. One important feature of our characterization of the microsatellites is that the related genes of the microsatellites were also identified. Together with the transcriptome resources generated here, these microsatellite loci provide a further valuable resource for future in-depth studies of functional genomics and ecology of black grouse.

## 5. Conclusions

The availability of new economical sequencing technologies has increased our capability to forge new links between genotypes and phenotypes in non-model systems. Such links may further inform on the relationships between organismal genotypes and the relative fitness of organisms in the environment, contributing to conservation efforts and a better understanding of ecologically important species. In this study, we performed transcriptome sequencing of spleen tissue in the black grouse and identified 7762 genes, 308 of which were estimated to be directly related to immune function. We also identified 1300 EST-microsatellites, designed primers for 526 of them and screened a subset for variations across populations. Collectively, these resources provide an excellent platform for investigating ecologically relevant genetic variations in black grouse populations. Transcriptome sequencing studies, such as this, provide an ideal first step towards assembling baseline knowledge of fitness-related genes, which may then be used to understand the variation and relevance of these genes in natural populations.

## Supplementary Material

Microsatellites with flanking primers and annotation information

## Supplementary Material

Microsatellites tested
